# Allergen Immunotherapy in an HIV+ Patient with Allergic Fungal Rhinosinusitis

**DOI:** 10.1155/2015/875260

**Published:** 2015-04-14

**Authors:** Ian A. Myles, Satyen Gada

**Affiliations:** ^1^Bacterial Pathogenesis Unit, Laboratory of Clinical Infectious Diseases, National Institute of Allergy and Infectious Diseases, National Institutes of Health, 9000 Rockville Pike, Building 33, Room 2W10A, Bethesda, MD 20892, USA; ^2^Allergy/Immunology/Immunization Service, Department of Medicine, Walter Reed National Military Medical Center, 8901 Wisconsin Avenue, Bethesda, MD 20889, USA

## Abstract

Patients with HIV/AIDS can present with multiple types of fungal rhinosinusitis, fungal balls, granulomatous invasive fungal rhinosinusitis, acute or chronic invasive fungal rhinosinusitis, or allergic fungal rhinosinusitis (AFRS). Given the variable spectrum of immune status and susceptibility to severe infection from opportunistic pathogens it is extremely important that clinicians distinguish aggressive fungal invasive fungal disease from the much milder forms such as AFRS. Here we describe a patient with HIV and AFRS to both remind providers of the importance of ruling out invasive fungal disease and outline the other unique features of fungal sinusitis treatment in the HIV-positive population. Additionally we discuss the evidence for and against use of allergen immunotherapy (AIT) for fungal disease in general, as well as the evidence for AIT in the HIV population.

## 1. Introduction

A recent review by Callejas and Douglas outlined five major types of fungal rhinosinusitis [1]. Fungal balls are dense growths mainly of* Aspergillus* species that afflict mostly middle-aged to elderly women; the disorder can occur in both immunocompromised and immunocompetent hosts and has excellent surgical cure rates. Another potential fungal sinus disease found in immunocompetent hosts is the far less common granulomatous invasive fungal rhinosinusitis. This disorder is geographically limited mostly to Sudan, India, Pakistan, and Saudi Arabia, most often caused by* Aspergillus flavus*, and is treated with a combination of surgery and itraconazole. Immunosuppressed patients may present with acute or chronic invasive fungal rhinosinusitis, with the distinguishing feature being the severity of the immunologic defect. In chronic invasive disease, the defect tends to be subtle such as the immune dysfunction seen in poorly controlled diabetics and patients on low-dose corticosteroids. In contrast, the acute form tends to manifest in patients with severe reduction in neutrophil numbers or function, such as patients undergoing chemotherapy or afflicted with blood-based cancers. Chronic invasive fungal rhinosinusitis is considered a medical emergency due to the potential for rapid invasion into the central nervous system imparting a high mortality rate.* Aspergillus* and* Mucorales* are the most common causative agents identified in both acute and chronic invasive diseases, and both forms are treated with immune reconstitution (if possible), surgical debridement, and systemic antifungals. Allergic fungal rhinosinusitis (AFRS) is also most often caused by* Aspergillus* species but may be due to* Alternaria*,* Bipolaris*, or* Curvularia* species. Atopic yet immunocompetent patients often present with this disorder and treatment includes surgery, nasal steroids, and perhaps the addition of allergen immunotherapy (AIT). Given the variable spectrum of immune status and susceptibility to severe infection from opportunistic pathogens, patients with HIV/AIDS can present with any of the five types of fungal rhinosinusitis. It is thus extremely important when seeing a patient with HIV and fungal sinus disease to distinguish aggressive fungal disease such as acute invasive fungal rhinosinusitis from the much milder forms such as AFRS (Table 1). Here we describe a patient with HIV and AFRS to remind providers of the importance of ruling out invasive fungal disease and other unique features of fungal sinusitis treatment in the HIV-positive population. Additionally we discuss the evidence for and against use of AIT for fungal disease in general, as well as the evidence for AIT in the HIV population.

## 2. Case Presentation

A 40-year-old male presented for evaluation with 15–20 years of congestion, headache, and anosmia. He reported a variable response to nasal steroids and oral antihistamines. In 1994, he underwent sinus surgery for sinus polyposis, after which his symptoms improved. Despite continued treatment with daily loratadine and then later fexofenadine, he required repeat sinus surgery in 2000. He was then placed on cetirizine with improved and stable symptoms for several months. However, around this time he was found to be HIV-positive and cetirizine was discontinued due to concern for drug interaction with his antiretroviral medications. Given continued symptoms and limited oral treatment options due to lack of efficacy or potential medication interactions, he was treated with allergen immunotherapy (AIT) from 2000 to 2003 to molds and dust mite per self-report. While he described symptomatic improvement on AIT, his treatment was interrupted after moving and was not restarted. After reporting worsening of symptoms including chronic nasal congestion with acute episodes of worsening when exposed to “moldy rooms” and cats, a repeat CT demonstrated evidence of recurrent pansinusitis. Subsequent MRI confirmed CT findings of pansinusitis with no evidence of invasive disease, prompting a third sinus surgery in late 2009. Thick, inspissated “allergic” mucin and nasal polyposis were noted intraoperatively, consistent with the diagnosis of AFS. Tissue examination revealed fungal elements by GMS/PAS stain and numerous eosinophils with growth of* Curvularia* on culture. His symptoms included stable, daily nasal congestion with exacerbations. The recurrent nature of his allergic fungal sinusitis raised the question of whether he would be best treated with reinitiation AIT, and thus he was referred to our allergy clinic for evaluation.

His past medical history was positive for adult attention deficit hyperactivity disorder and HIV with a CD4 count of 397 and an undetectable viral load. He denied any respiratory complaints and had no history of asthma. Vital signs were within normal limits. Physical exam was significant for surgical removal of much of the middle turbinates and dry nasal mucosa without erythema, edema, or nasal polyposis. There was no sinus tenderness. His medications included daily lamivudine, abacavir, raltegravir, efavirenz, methylphenidate, calcium supplements, budesonide suspension used in conjunction with nasal sinus saline rinse, azelastine, and mometasone nasal sprays. He had no history of ever receiving antifungal medications.

Our evaluation revealed negative skin prick testing to all relevant trees and grasses, as well as cat, dog, cockroach, and dust mite. He had positive skin prick testing to* Curvularia spicifera, Alternaria tenuis, Helminthosporium mix, Penicillium notatum*, and ragweed mix. Intradermal skin tests were positive to* Aspergillus fumigatus, Cladosporium mix*, and* Epicoccum nigrum*. Total serum immunoglobulin E (IgE) was 298.2 mg/dL. Serum sensitization was confirmed for* Cladosporium herbarum*,* Alternaria*, and* Aspergillus*. He had mild specific IgE elevations to ragweed and elder marsh. Specific IgE to grasses (Bermuda, Johnson, Kentucky Blue), elm, juniper, oak, olive tree, dust mite, cat, dog, and cockroach were all negative. Having established clinically relevant fungal sensitization and after discussing risks and potential benefits, AIT against the sensitized fungal allergens was initiated. He tolerated AIT without serious reactions, achieving maintenance after a one-year period of buildup (Table 2). At that time, repeat CT of sinuses revealed stable postoperative changes with no finding of recurrent fungal disease. He continued on maintenance AIT for two years and then discontinued due to his work schedule preventing compliance with monthly maintenance injections. He has remained on daily mometasone nasal spray as well as budesonide suspension with saline sinus lavage. Despite only two years of maintenance AIT, he has remained stable, with minimal symptoms and a recent endoscopy demonstrated only small, asymptomatic polyps.

## 3. Discussion

This case highlights the unique issues related to fungal sinus disease. Although this patient's clinical course ended up to be one of the successful treatments, as soon as fungal sinus disease is suspected in an immunocompromised patient, invasive disease should be excluded immediately, especially if neutropenia is also present [2, 3]. Bone erosion and extrasinus extension do not manifest until late in the course of invasive disease, and thus lack of such findings should not dissuade further work-up [4]. In one study the most suggestive (albeit nonspecific) finding in HIV patients with invasive disease was severe unilateral mucosal thickening [5].

Overall, up to 68% of HIV patients report some form of sinus disease [4, 6]. Part of the pathogenesis may be reduced ciliary clearance seen in HIV-positive individuals [7] perhaps leading to mucus stasis [4]. While the evaluation of sinus disease in the HIV-positive population is often similar to seronegative patients, in addition to ruling out invasive disease clinicians should ensure that any sinus cultures are sent for all possible cultures including fungal, viral, aerobic, anaerobic, and mycobacterial ones [4]. The most common pathogens causing acute sinusitis in the HIV-positive population are* S. pneumoniae*,* H. influenza*,* M. catarrhalis*, and* S. viridans*, whereas chronic sinus disease in HIV-positive individuals is most often* S. aureus*,* S. epidermidis*, or anaerobic bacteria [1, 4]. When invasive fungal disease is encountered, clinicians must understand that mortality is high and thus aggressive, and open surgical debridement with adjunct liposomal amphotericin B is indicated [3].

In contrast with invasive disease, allergic fungal sinusitis in patients with HIV is addressed identically to seronegative patients. Diagnosis may involve the Bent III and Kuhn criteria [8]: (1) nasal polyps, (2) sinus content positive for fungi on staining and/or cultures, (3) eosinophil rich mucin, (4) no evidence of invasive disease but positive CT findings of sinus expansion or opacification, and (5) evidence of allergic sensitivity in either skin testing or serum IgE specific to fungi (with skin testing showing greater sensitivity than serum IgE testing [1]). Unlike our patient, total IgE is typically elevated over 1000 IU/mL; however, unlike in a similar disease ABPA (allergic bronchopulmonary aspergillosis) following IgE levels does not appear to provide clinical benefit [1]. Treatment involves nasal saline irrigation, nasal corticosteroids, and consideration for short-courses of systemic corticosteroids [1, 9–12]. Of note, our patient's systemic antihistamines were discontinued at the time of his HIV diagnosis yet while antiretroviral medications carry a general interaction cytochrome p450 warning with antihistamines (presumably the reason for the discontinuation), the specific interactions are described for H-1 antagonists such as astemizole and there are no contraindications to H-2 receptor blocker use in the presence of antiretroviral therapy [13]. There are conflicting recommendations for use of systemic antifungals in AFRS [9, 14]; however one recent study reported significant reduction in recurrence of AFRS with postoperative use of intranasal fluconazole [9]. Surgical treatment involving functional endoscopic sinus surgery (FESS) may also be indicated; however patients should be informed that recurrence is high even with surgical intervention [15, 16] and many patients require several surgeries before clinical resolution is obtained [17, 18].

This case also highlights another potential therapeutic intervention, one with far less clarity on utility, allergen immunotherapy. The use of AIT in allergic fungal sinusitis started on shaky ground when a study of 7 patients reported that only 2 patients improved while 5 had worsening of symptoms [19, 20]. Furthermore, the researchers uncovered induction of fungal-specific IgG, raising the possibility of inducing a type III immune-complex reaction [20]. However, further investigation revealed that the two patients that showed improvement started AIT after surgery, whereas the five that worsened started prior to their operation [20, 21] and further investigation has failed to show any signs of the theorized immune-complex induction [21]. A recent review recommends waiting 4–6 weeks after surgery to begin AIT, surmising that AIT may worsen symptoms in those with active disease but may aide those in a disease-free postsurgical window [20]. Other early reports of treatment failure were subsequently shown to have been using end-point concentrations of antigens far below what would be recommended in modern AIT and thus may be explained simply by dose limitations [21]. The largest evaluation, a retrospective evaluation of 36 post-FESS patients treated with AIT compared to 24 controls, found that AIT imparted a threefold reduction in the need for repeat surgery (11% versus 33%) at a mean follow-up of about four years [17]. In a clinical trial including 22 patients' status after FESS, the 11 subjects that received AIT had significantly better outcomes on quality of life surveys, endoscopic mucosal staging, and reduced use of systemic and nasal steroids at their mean follow-up of 33 months [22]; however, this study did not include a placebo control. Overall, these studies and others examining AIT for allergic fungal sinus disease show promise when correctly timed and targeted; however much work is still needed to elucidate the optimal timing, dose, and duration.

Given that our patient had a personal history of benefit from AIT we elected to resume treatment and will need to monitor despite little literature guidance on duration. However, the literature does not offer any better data for pollen and venom AIT in HIV-positive patients than what it offers for fungal sinus disease. In addition, although the recommendation for AIT is 3–5 years when treating allergic rhinitis and allergic asthma, there are no specific recommendations for length of treatment in AFS. A potential, yet theoretical, complication of AIT in HIV-positive patients would be the expansion of memory CD4+ T cells and a subsequent increase in viral proliferation [23, 24]. Yet one case report [25] and another care series [26] have described a total of four patients treated for periods ranging from 24 to 102 weeks, and none showed any evidence of either increased viral load or instability in their CD4+ counts. Despite this evidence of benefit, a survey of providers revealed that when treating HIV-positive patients, 26% would not perform skin testing and 60% were less likely to treat with AIT due to seropositivity [27, 28]. While we lack comparative controls, treatment in our patient for allergic sinus disease with 24 months of maintenance AIT was well tolerated and demonstrated benefit, adding to the case reports of safe AIT treatment in patients with HIV.

## Figures and Tables

**Table 1:** Review of sinus disease categories and features.

Sinus diseases of immunocompetent hosts				
Type	Pathogen	Host features	Treatment	Pearls

Fungal ball	*Aspergillus* species	Females over ~50 years of age	Outpatient surgery	High cure rate
Granulomatous invasive fungal rhinosinusitis	*Aspergillus flavus *	All demographics, in Sudan, India, Pakistan, and Saudi Arabia	Outpatient surgery and systemic antifungals	Postoperative itraconazole may reduce relapse rate
Allergic fungal rhinosinusitis	*Aspergillus* sp., *Alternaria* sp., *Bipolaris* sp., and *Curvularia* sp.	Atopic patient	Outpatient surgery, allergic treatments (nasal steroids), and considering allergen immunotherapy	Best evidence is for allergen immunotherapy initiation 4–6 weeks after surgery

Sinus diseases limited to immunocompromised hosts				
Type	Pathogen	Host features	Treatment	Pearls

Acute invasive fungal rhinosinusitis	*Aspergillus* species, *Rhizopus* sp., and *Mucor* sp.	Reduced neutrophil number of functions, HIV	Inpatient surgery, systemic antifungals, and immune reconstitution	Must be distinguished from noninvasive disease in immunocompromised host, high mortality
Chronic invasive fungal rhinosinusitis	*Aspergillus* species, *Rhizopus* sp., and *Mucor* sp.	Less severe impairment such as diabetes, systemic corticosteroids, and HIV	Outpatient surgery, systemic antifungals, and immune reconstitution	Must be distinguished from noninvasive disease in immunocompromised host; recurrence is possible

Modified from Callejas and Douglas, 2013 [1].

**Table 2:** Maintenance allergy immune-therapy dose composition for our presented case report.

Species	Concentration (weight/volume)	Volume
*Alternaria *	1 : 20	0.5 mL
*Aspergillus fumigatus *	1 : 20	0.5 mL
*Helminthosporium *	1 : 10	0.5 mL
*Hormodendrum/Cladosporium *	1 : 20	0.5 mL
*Curvularia *	1 : 20	0.5 mL
*Epicoccum *	1 : 40	0.5 mL
*Penicillium *	1 : 20	0.5 mL
Diluent	—	6.5 mL
Total	1 : 200	10 mL
